# Potential new mechanisms of pro-arrhythmia in arrhythmogenic cardiomyopathy: focus on calcium sensitive pathways

**DOI:** 10.1007/s12471-017-0946-7

**Published:** 2017-01-19

**Authors:** C. J. M. van Opbergen, M. Delmar, T. A. B. van Veen

**Affiliations:** 1 Department of Medical Physiology, Division of Heart & Lungs, University Medical Center Utrecht, Utrecht, The Netherlands; 2 The Leon H. Charney Division of Cardiology, New York University School of Medicine, New York, USA

**Keywords:** Arrhythmogenic cardiomyopathy, Calcium, Arrhythmia, CaMKII, Phospholamban

## Abstract

Arrhythmogenic cardiomyopathy, or its most well-known subform arrhythmogenic right ventricular cardiomyopathy (ARVC), is a cardiac disease mainly characterised by a gradual replacement of the myocardial mass by fibrous and fatty tissue, leading to dilatation of the ventricular wall, arrhythmias and progression towards heart failure. ARVC is commonly regarded as a disease of the intercalated disk in which mutations in desmosomal proteins are an important causative factor. Interestingly, the Dutch founder mutation *PLN R14Del* has been identified to play an additional, and major, role in ARVC patients within the Netherlands. This is remarkable since the phospholamban (PLN) protein plays a leading role in regulation of the sarcoplasmic reticulum calcium load rather than in the establishment of intercellular integrity. In this review we outline the intracellular cardiac calcium dynamics and relate pathophysiological signalling, induced by disturbed calcium handling, with activation of calmodulin dependent kinase II (CaMKII) and calcineurin A (CnA). We postulate a thus far unrecognised role for Ca^2+^ sensitive signalling proteins in maladaptive remodelling of the macromolecular protein complex that forms the intercalated disk, during pro-arrhythmic remodelling of the heart.

## Genetic causes of cardiomyopathy

Over the last decades, multiple studies have identified over 100 genes and mutations involved in various forms of cardiac disease such as hypertrophic, dilated, and arrhythmogenic cardiomyopathy (HCM, DCM, and ACM, respectively) [1]. These genes mainly encode proteins of the contractile machinery, but also proteins that participate in calcium homeostasis and ones that reside in the intercalated disk (ID). As a consequence, mutations in these genes have pathogenic effects on several cardiac cellular functions, such as contractile force generation, force regulation, signal transduction, excitability and intracellular calcium handling. Since the discovery of the first causative mutations for cardiomyopathies, attention for such inherited genetic disorders has greatly increased. To uncover the consequences of these genetic mutations contributes importantly to knowledge of the underlying pathogenic mechanisms and has introduced a regimen of clinical genetic evaluation of affected individuals and their relatives. Early identification of asymptomatic mutation carriers, via genetic screening, is of major prognostic importance since cardiomyopathies, with an emphasis on ACM, can lead to sudden cardiac death as a result of malignant ventricular arrhythmias even before the onset of any symptoms.

Interestingly, there are multiple genes in which genetic aberrancies cause different types of cardiomyopathies and even opposite functional effects are present. For example, mutations in proteins involved in calcium handling, such as phospholamban (PLN), which mostly correlate to DCM also manifest in the setting of ACM [2]. On the other hand, mutations in the gene encoding the desmosomal protein plakophilin2 (PKP2) are classically correlated with ARVC (a subform of ACM), [3] but can additionally be identified in DCM patients [4]. Likewise, mutations in *PKP2* are also present in patients suffering from Brugada syndrome, a disease commonly related to mutations in multiple ion channels instead of desmosomal proteins [5]. These studies suggest that distinct cardiac proteins should not be considered to be completely autonomous working entities, but rather that they function in different macromolecular protein complexes. In this review we will focus specifically on gene mutations that relate to ACM and the potential mechanisms that drive proarrhythmic cardiac remodelling in this disease. For a subset of these mutations, particularly those involving PLN, we postulate a potential role for calcium sensitive signalling pathways.

## Pathophysiological remodelling in arrhythmogenic cardiomyopathy

During cardiomyopathy, not exclusively limited to ARVC, ventricular arrhythmias can arise out of electrical and structural cardiac remodelling, which disrupts cell-cell communication, excitability and the pump function of the heart [6]. Electrical remodelling is based on abnormal expression, distribution and regulation of ion channels and gap junctions, and a heterogeneous expansion of the extracellular matrix (fibrosis formation) which results in a disturbed electrical cell-cell signalling between cardiomyocytes or between cardiomyocytes and (myo)fibroblasts [6, 7]. Increased pathophysiological intracellular calcium concentrations, caused by dysfunction of calcium regulatory proteins, negatively affects the contraction and relaxation dynamics of the heart (reviewed by ‘Qu et al. 2015’) [6]. Structural cardiac remodelling involves myocyte death, fibre disorders, changes in cell size (hypertrophy), and collagen deposition (fibrosis). Structural remodelling disturbs the impulse propagation between cardiomyocytes and hampers pump function of the entire heart [6].

ARVC, the most familiar form of ACM, is a cardiac disease mainly characterised by a gradual replacement of the myocardial mass by fibrous and fatty tissue, which causes dilatation of the ventricular wall and progression towards heart failure. As such, this is classically defined as structural remodelling, but it also includes a disruption of proper electrical signalling, with a high susceptibility for ventricular arrhythmias. As described in several studies, remodelling also results in a combination of reduced sodium channel (Na_v_1.5), gap junction channels (constituted of connexin43, Cx43) and plakoglobin localisation at the ID of patients [8]. ARVC is a familial disease with an estimated frequency of 1:5000 people all over the world, though in some regions prevalence is more pronounced [9]. In about 60% of the ARVC cases, a mutation is the underlying cause of the disease [2]. Mutations in genes encoding the desmosomal proteins PKP2, plakoglobin (gene name *JUP*), desmoplakin (DSP), desmocollin-2 (DSC2) and desmoglein-2 (DSG2), are highly related to development of ARVC [1]. Those mutations are predominantly found in the *PKP-2* gene, but have also been discovered in genes encoding non-desmosomal proteins such as ryanodine receptor2 (RYR2), transforming growth factor β3 (TGFβ3), lamin A/C (LMNA), transmembrane protein 43 (TMEM43), desmin (DES), titin (TTN), αT-catenin (CTNNA3) and PLN [1].

The fact that mutations in genes encoding desmosomal and non-desmosomal proteins commonly lead to a comparable clinical phenotype suggests that more than one underlying mechanism could be involved in the process of cardiac remodelling in ARVC patients. This is strengthened by the identification of a role for mutations in the sarcoplasmic reticulum (SR) associated proteins PLN and RYR2, and the fact that in the other 40% of patients diagnosed with ARVC, no causative mutations, or mutations not yet linked to ARVC, have been detected thus far [1]. PLN is a transmembrane SR phosphoprotein and a key regulator of calcium homeostasis. As expected, mutations in *PLN* mostly lead to inhibition of the calcium re-uptake into the SR [10]. Interestingly, next to the classical desmosomal mutations that trigger instability of the ID (potentially due to trafficking defects), the pathogenic *PLN-R14Del *mutation is identified in patients diagnosed with ARVC. In such patients, a differential diagnosis of DCM or ARVC can be made [2]. In the Netherlands, the *PLN-R14Del* mutation is a founder mutation and has been identified in 10–15% of patients diagnosed with either DCM or ARVC [11]. This similarity in pathophysiological remodelling driven by dysfunction/mislocalisation of proteins in the ID and proteins related to the intracellular calcium handling may perhaps not be as long distance as it seems at first glance. Present studies provide an interesting new view into the possible interaction between structures classically defined as separate (ID and calcium homeostasis). Next to the well-known classical model, based on malfunctioning/mislocalised desmosomal proteins, also involvement of alternative mechanisms underlying ARVC phenotype should be mentioned. The canonical Wnt/β-catenin signalling pathway and hippo pathway illustrate pathways which are affected by ID proteins under pathogenic conditions, ending in recapitulation of the ARVC phenotype [12, 13]. In this regard, the common observation that plakoglobin signals are reduced or even absent at the ID is important [8, 14], while glycogen synthase kinase 3β (GSK3β) seems to accumulate at the ID during pathological remodelling [15].

## Cross talk within the intercalated disk

To withstand the strong and frequent mechanical forces of the beating heart, individual cardiomyocytes tightly connect to each other via a subcellular domain named the ID [14]. IDs are of major physiological importance as they not only physically connect cardiomyocytes by means of desmosomes and adherence junctions, but also couple cardiomyocytes electrically to ensure fast propagation of electrical signals, through gap junctions [14]. Gap junctions are agglomerates of multiple intercellular channels that consist of two connexons (hemi channels), which are engaging structures composed of hexametric clusters of connexin proteins, delivered by cells on both sides of each junction. In the working myocardium, these channels are predominantly composed of connexin40 (Cx40, atria) and Cx43 (atria and ventricles). Electrical coupling depends on the organisation of gap junctions at the ID, whose abundance, resistivity and distribution defines the characteristics of electrical impulse propagation between cardiomyocytes. The classical outlook of the arrangement at the ID with its separated structures such as desmosomes, gap junctions, ion channels and connexons, is currently changing. Knowledge about the relation between different structures of the ID is expanding in detail and it is becoming more and more clear that the ID in fact is a complex and sophisticated protein network.

Accumulating experimental evidence supports the idea that the ID should be considered to be a singular arranged functional entity in which macromolecular complexes mechanically and electrically interact in synchrony [16]. This is exampled by the fact that the loss of desmosomal integrity, caused by PKP2 dysfunction, leads to a reduced sodium current (*I*_Na_), aiding an interaction between the desmosome and sodium channel complex [5]. This interaction could partly be related to disturbed trafficking of proteins to the ID. A study by Sato et al. presented colocalisation of Na_v_1.5 (the alpha-subunit of the sodium channel) with PKP2 [17], whereas a very recent study of the same group identified colocalisation of these sodium channels with N‑cadherin, a protein of the adherence junction [16]. When PKP2 expression was decreased through RNA silencing in neonatal rat ventricular myocytes, this also resulted in a decrease of the total Cx43 content and a significant redistribution of Cx43 to the intracellular space. Multiple experiments showed that Cx43 and PKP2 coexist in the same macromolecular complex. Besides this, ankyrin-G (encoded by *ANK3*) shows to be a key component of linkage within the ID as it is related to three complexes often considered independent: the voltage-gated sodium channel, gap junctions, and the cardiac desmosome [17, 18]. The intermolecular interactions between proteins relevant to mechanical junctions, and those that constitute gap junctions/sodium channels embeds important implications for the pathophysiology of inherited arrhythmias, such as in ACM [17]. This specifically accounts for patients in which the ACM is caused by mutations in desmosomal proteins. However, it is more complex to understand why patients show a similar clinical phenotype when causative mutations are present in non-desmosomal proteins such as PLN, a protein heavily involved in calcium homeostasis. Certainly if we bear in mind that also in these patients pro-arrhythmic remodelling of the ID is reported [11, 19].

## Calcium dynamics and pathophysiological signalling

PLN plays a leading role in cardiac excitation contraction coupling which relies on tightly regulated calcium fluxes that are triggered when an action potential enters from adjacent cells. Voltage gated L‑type calcium channels (LTCC) on the cell membrane of striated muscle cells open and calcium enters the cell. This influx of calcium into the cell subsequently triggers opening of RYR2, leading to a coordinated efflux of calcium out of the SR. Local calcium peaks result in binding of calcium ions to the troponin complex, which consists of three regulatory proteins that form a contractile complex. If calcium ions unbind from troponin the myofilaments are able to relax again. Calcium is removed out of the cardiomyocytes by Na^+^/Ca^2+^ exchange channels (NCX) at the cell membrane or stored back in the SR by sarco/endoplasmic reticulum Ca^2+^-ATPase channels (SERCA, reviewed by ‘Luo et al., 2013’) [20]. The open and closed state of gap junction/ion channels and channels involved in calcium handling is tightly controlled by phosphorylation and dephosphorylation, through kinases and phosphatases. Under physiological conditions their activation is mainly regulated by protein kinase A (PKA), protein kinase C (PKC), and mitogen-activated protein kinase signalling. Downstream activity of protein kinases such as PKA and PKC regulates Ca^2+^ homeostasis by phosphorylation of Ca^2+^ channels, such as LTCC, SERCA, and RYR2. This regulation is important to adapt to changing demands as, for example, an elevated cardiac output. Dephosphorylated PLN interacts with SERCA and inhibits Ca^2+^ pump activity into the SR [10]. By phosphorylation of PLN upon β‑adrenergic stimulation and enhanced cyclic AMP-dependent protein kinase A (PKA) activity, the inhibitory effect of PLN on SERCA is relieved. This leads to increased initial rates of SR Ca^2+^ uptake, accelerated relaxation, enhanced SR Ca^2+^ load and increased Ca^2+^ release by RYR2 during systole [21].

Under pathophysiological conditions, functional control of cellular excitability and excitation-contraction coupling shifts from kinases such as PKA and PKC towards calmodulin dependent kinase II (CaMKII) and calcineurin A (CnA) [22, 23]. Under pathological conditions the diastolic calcium levels can become persistently higher, which in the end compromises pump function through insufficient relaxation and a reduced force of contraction due to lower calcium availability during systole [20]. Diastolic calcium concentrations do not solely rely on LTCC and SR Ca^2+^ regulation, since alterations in the kinetics of sodium channels (triggering late sodium current, *I*_Na-L_), NCX and PLN activity are also of importance [10, 24]. An increase in the amplitude of *I*_Na-L_ prolongs the action potential duration, increases the transmural dispersion of repolarisation which triggers arrhythmogenesis [25]. An increase in *I*_Na-L_ also increases the intracellular sodium concentration and subsequently raises [Ca^2+^]_i_ via the reverse-mode NCX [26].

**Fig. 1:**
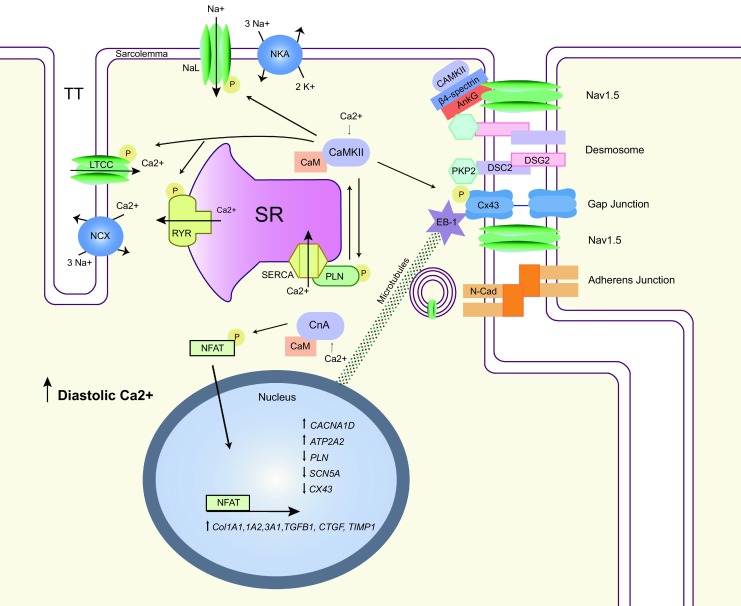
Overview illustrating the pro-arrhythmic interactions between Ca^2+^-sensitive proteins, intercalated disk proteins and ion channels. *SR* sarcoplasmatic reticulum, *RyR* ryanodine receptor, *SERCA* SR Ca^2+^ ATPase, *PLN* phospholamban, *LTCC* L-type Ca^2+^ channel, *NaL* late sodium current, *NCX* Na^+^/Ca^2+^ exchanger, *NKA* Na^+^/K^+^-ATPase, *AnkG* ankyrin-G, *DSC2/DSG2* desmocolin-2/desmoglein-2, *PKP2* plakophilin-2, *Cx43* connexin43, *N-Cad* N-Cadherin, *CaMKII* Ca^2+^/calmodulin-dependent protein kinase II, *CaM* calmodulin, *CnA* calcineurin A, *EB-1* end-binding protein 1, *NFAT* nuclear factor of activated T‑cells, *CACNA1D* voltage-dependent L‑type calcium channel subunit alpha-1D, *ATP2A2* sarcoplasmic/endoplasmic reticulum calcium ATPase 2, *SCN5A* sodium channel protein type 5 subunit alpha, *Col1A1/1A2/3A1* alpha-1 type I/1 type 2/3 type 1 collagen, *TGFβ1* transforming growth factor beta 1, *CTGF* connective tissue growth factor, *TIMP1* metallopeptidase inhibitor 1

Besides affecting kinases and phosphatases such as CaMKII and CnA, the intracellular calcium concentration is also capable of affecting the ID structure directly. Although it is known that elevating intracellular calcium conditions from very low towards the physiological state causes targeting and maturation of desmosomes from the endoplasmic reticulum to the cellular membrane [27], currently it is still unknown whether such influences can also be triggered by a persistently elevated diastolic calcium level above the physiological concentration. Importantly, the signal transduction pathways induced under persistently elevated calcium levels further trigger cardiac remodelling and electrical and contractile modifications [24]. In the following we propose a link between increased diastolic Ca^2+^ concentrations, activation of CaMKII and CnA, and electrical and structural cardiac remodelling in the setting of ACM (Fig. 1).

## Phospholamban dysfunction

Deregulated intracellular Ca^2+^ handling in cardiomyocytes is frequently attributed to impaired SR functioning, leading to heart failure, in which dysfunction of the different key proteins involved can each be causative. Dysfunction of PLN has been reported to play a key role in cardiac pathophysiology and up to now several different mutations have been identified in patients [21]. Characterisation of those *PLN* mutations generally revealed disruption of intracellular calcium handling, precipitating clinical manifestations of ACM, DCM and heart failure (see Table 1).

Of special attention in this review is the *PLN-R14Del* mutation since this mutation is known to trigger development of ACM in patients [19]. PLN is a phosphoprotein which, when dephosphorylated, inhibits the Ca^2+^ affinity of SERCA. PLN phosphorylation can take place at the Ser16 by PKA (during sympathetic stimulation) or at Thr17 by CaMKII (predominantly during pathophysiological conditions), which reverse the inhibitory effect on SERCA [28]. The *PLN-R14Del* mutation is associated with the deletion of the highly conserved basic amino acid Arg-14 in the coding region. As a result, phosphorylation is still possible but the inhibitory effect is no longer relieved. Coexpression of wild-type PLN and mutant PLN-R14Del, in human embryonic kidney (HEK) cells confirmed the super inhibition of the SERCA channel [10]. This super inhibitory effect is likely caused by partial destabilisation of the PLN pentamer, leading to production of highly inhibitory monomers and a persistent PLN-SERCA association. The dominant inhibitory effect of this mutant was not alleviated by phosphorylation through PKA, leading to ventricular dilatation, contractile dysfunction and ventricular arrhythmias. Transgenic mice, overexpressing the PLN-R14Del protein, recapitulated the human cardiomyopathy and resulted in premature death. Next to the disturbance in Ca^2+^ transients, PLN-R14Del patients also show classical ARVC-like features as fibrofatty replacement and myocardial disarrangement [10]. A study in endomyocardial samples taken from PLN-R14Del patients showed depressed or absent plakoglobin levels indicative of remodelling of the ID in 71% of the cases [11].

In addition to abnormalities in Ca^2+^ transients and electrical instability, in cardiomyocytes derived from human induced pluripotent stem (iPS) cells that were generated from patients bearing the *PLN-R14Del* mutation, an abnormal distribution of the mutated protein towards the cytoplasm was reported [29]. Knocking down the mutated *PLN* gene and simultaneous overexpression of a normal *PLN* gene (genetic repair) appeared able to reverse this pathological phenotype *in vitro*. In a different experimental mouse model, the mutation was introduced into PLN null mice, to generate a homozygous PLN-R14Del knockout model [21]. Comparable with the situation in PLN-L39stop mutants, colocalisation of PLN with SERCA was lost and no inhibition on SR Ca^2+^ transport and contractility in these PLN-R14Del mice was present. In contrast, PLN-R14Del mutated protein misrouted towards the sarcolemmal Na^+^/K^+^-ATPase (NKA) in the plasma membrane and stimulated its activity. Eventually these PLN-R14Del mice showed cardiac remodelling in terms of ventricular dilation and interstitial fibrosis [21].

The missense mutation at PLN residue 9 (*PLN-R9C*) has been reported to cause inherited DCM and heart failure in patients [30]. Studies in transgenic PLN-R9C mice showed that the mutated protein did not impair Ca^2+^ transients by direct inhibition of SERCA, rather than it depended on trapping of PKA by the mutant protein that resulted in a blocked PKA-mediated phosphorylation of wild-type PLN. This finally delayed intracellular calcium transients through super inhibition of SERCA [30]. A different T116G point mutation resulting in PLN-Leu-39-stop (*PLN-L39stop*) was also discovered in familial cases of heart failure [31]. Whereas the heterozygous mutation caused cardiac hypertrophy, homozygous patients developed lethal DCM and heart failure. Homozygous hearts showed a 50% reduction of PLN mRNA while PLN protein diminished to a negligible level suggesting that disturbed calcium handling could not be causative for the disease. Surprisingly, homozygous patients demonstrated extensive fibrofatty replacement and myocardial disarrangements. In vitro, adenoviral transfection of recombinant *PLN-L39stop* in adult rat cardiomyocytes indeed confirmed the absence of a maladaptive effect on SERCA and contractility. Viral transfection of *PLN-L39stop* in HEK cells revealed destabilised PLN expression and redirected minor amounts of PLN towards the cytosol or plasma membranes. This study emphasised a clear contrast between the disease phenotypes in human and rodents since ablation of PLN in mice seemed to be beneficial in the setting of heart failure, whereas human carriers of *PLN-L39stop* develop severe and lethal cardiomyopathies [31]**.**

Very recently it was shown that overexpression of PLN-R25C in adult rat cardiomyocytes extremely suppressed the affinity of SERCA for Ca^2+^, resulting in a decreased SR Ca^2+^ concentration and Ca^2+^ transients [32]. Interestingly, this super inhibition of SERCA caused elevated diastolic Ca^2+^ concentrations and CaMKII activation. CaMKII activation subsequently enhanced RYR2 phosphorylation and leakage of Ca^2+^ out of the SR, ending in an impaired cardiac contractility and ventricular arrhythmias. To further stress the pivotal role for CaMKII in this pathophysiological remodelling, KN93 inhibition of CaMKII abolished the PLN-R25C triggered Ca^2+^ sparks [32].

In general, dysfunctional PLN highly disrupts the cardiac intracellular calcium handling, finally ending in hypertrophy, DCM, ventricular arrhythmias, heart failure and premature death. Mainly SERCA, RYR2 and the SR load are affected, leading to increased diastolic calcium concentrations and decreased Ca^2+^ transients. In spite of the disturbed calcium homeostasis a subgroup of PLN-R14Del patients present an ARVC-like phenotype, resembled by fibro-fatty replacement and ID disarrangement. Comparable alterations were also reported in patients with a different PLN mutation, such as *PLN-L39stop*. The most recent study also presented an increased CaMKII activation by PLN-R25C. Several studies in cardiomyocytes have already proven that increased diastolic Ca^2+^ concentrations cause CaMKII auto-phosphorylation and CaMKII activation [22, 33, 34]. Importantly, next to CaMKII, also the Ca^2+^/CaM activated phosphatase CnA is sensitive to increased diastolic calcium concentrations [23]. In that regard it is of interest to uncover if a pathophysiological activation of CaMKII and CnA in ACM patients potentially contributes to the adverse remodelling in ACM. Because CaMKII and CnA play a key role in electrical and structural remodelling of the heart, as postulated in the following paragraphs, it is not unlikely that also in this particular disease they modulate aspects of electrical signalling, excitation-contraction coupling and tissue architecture.

## CaMKII in cardiomyopathy

CaMKII and CnA are routinely present in the heart, but transcription, expression and activation of these proteins is enhanced during cardiomyopathy [35]. This phenomenon suggests a pathophysiological role for these proteins in the development of heart failure. Persistent activation of beta-adrenergic signalling pathways will activate CaMKII, but most important, phosphorylation of Ca^2+^ channels increases the diastolic intracellular Ca^2+^ concentration, which facilitates permanent activation of CaMKII via calmodulin (CaM). Moreover, CaMKII is able to maintain its activated state independent of calcium-activated CaM via autophosphorylation and via the influence of reactive oxygen species (reviewed by ‘Heineke et al. 2006’) [36]. Amongst many others, activated CaMKII phosphorylates cardiac proteins that increase inflammatory signalling and apoptosis [37]. A study in pneumocytes showed that activated CaMKII drives apoptosis which can lead to pulmonary fibrosis [38]. Chronic pressure overload in a murine knockout model of CaMKII resulted in a tempered increase in ventricular wall thickness and no significant levels of fibrosis [22]. Illustrative for its well-studied and maladaptive role in disturbance of calcium handling, a study in patients with heart failure suggested that pathophysiologically enhanced CaMKII levels in cardiomyocytes increased SR Ca^2+^ leak, which consequently depleted the SR as Ca^2+^ store, and decreased contractility [33]. A link of activated CaMKII to fatty replacement, one of the striking hallmarks of remodelling in ARVC hearts, currently is lacking.

## CaMKII remodelling of ID proteins

Beyond its detrimental role in SR calcium handling, CaMKII can also influence electrical signalling between cardiomyocytes at the level of the ID, by phosphorylation of ion channels such as the sodium channel. The CaMKII-δ isoform stably interacts with two looping domains of the main cardiac voltage-dependent sodium channel (constituted from Na_v_1.5 proteins) and facilitates phosphorylation at the Ser-516, Ser571 and Thr-594 site [39]. The pathological regulation of cardiac sodium channels by CaMKII is not well studied in human disease, but animal models of triggered heart failure presented independent upregulation of CaMKII levels and enhanced *I*_Na_ during cardiomyopathy [34]^.^ In dogs with chronic heart failure the *I*_Na-L_ and total sodium influx levels where enhanced by Ca^2+^/CaMKII signalling under physiological and pathophysiological conditions, which was manifested by slowing down of the inactivation kinetics of the channel [34]. In this model, the excess of sodium entry was counteracted by activation of the NCX, which in turn even further enhanced the already elevated diastolic calcium level. A recent study of Glynn et al. in mice revealed that phosphorylation of Na_v_1.5 at Ser571 regulates the increase in *I*_Na-L_, but not other channel properties previously linked to CaMKII [39]. Inhibition of CaMKII in mice resulted in down regulation of *I*_Na_, which negatively affected the sodium channels ability to depolarise the cell whereas phosphorylation of sodium channels in rabbit cardiomyocytes delayed the *I*_Na_ recovery after inactivation and enhance the *I*_Na-L_ [40]. The observations made in these different models indicate that enhanced activation of CaMKII increases the depolarising current, elongating the action potential and thereby increases the susceptibility to arrhythmias.

Conditional knockout of ankyrin-G in a mouse model showed that ankyrin-G targets Na_v_1.5 proteins and its regulatory protein CaMKII to the ID for which βIV-spectrin is necessary [18, 41]. Knockout of ankyrin-G resulted in a decreased Na_v_1.5 expression and membrane trafficking, and a concomitantly decreased *I*_Na_ and *I*_Na-L_, highlighting the dependence of the sodium channel on other protein partners in the ID that serve as a regulatory platform [18, 42]. Moreover, ankyrin-G links sodium channels with broader ID signalling nodes, as loss of ankyrin-G also resulted in reorganisation of PKP2 and lethal arrhythmias in response to β‑adrenergic stimulation [18]. A study by Xu et al. clarified that enhanced cellular calcium entry totally diminished the open probability of Cx43 gap junction channels which suggests that gating was affected by a Ca^2+^/CaM-dependent mechanism [43]. These results reveal that CaM already links towards gap junctions by itself and additionally suggest a relation between CaMKII and Cx43. This was recently confirmed in an *in vitro* study with cultured cardiomyocytes, where one novel serine residue in the Cx43 carboxyl terminus was identified that can be phosphorylated by CaMKII, which suggests that CaMKII may phosphorylate, and thereby regulates the activity of Cx43 channels in normal and diseased hearts [44].

## Role for calcineurin in fibrosis formation and conductional remodelling

Calcineurin is a serine-threonine dimeric phosphatase, consisting of the catalytic subunit CnA and the regulatory subunit calcineurin B (CnB). Next to the above described regulation of CaMKII, Ca^2+^ saturated CaM directly binds and activates CnA. Coherent to the fact that CaM needs to be activated by Ca^2+^, the amount of CaM needed for activation of CnA decreases by increasing intracellular calcium concentrations. Binding and dephosphorylation of nuclear factor of activated T‑cells (NFAT) transcription factors by CnA in the cytoplasm allows translocation of NFAT into the nucleus, which induces gene expression of adverse remodelling genes. Other kinases such as JNK, p38, GSK3β and PKA phosphorylate specific NFAT family members and block the nuclear translocation. (Reviewed by ‘Heineke et al*.* 2006’) [36].

Activity of CnA was significantly increased in the compensated and decompensated hypertrophic myocardium of patients with coronary artery disease, and it is suggested to play a major role in fibrosis formation [45]. Transgenic mice with a constitutively active form of CnA present in the myocardium display tremendous deposits of collagen in the myocardium and show trans-differentiation of ventricular fibroblasts into myofibroblasts, a phenotype known for its high profibrotic activity [23]. Mice overexpressing a constitutively active form of CnA presented pathological remodelling and impaired Cx43 protein levels. Fontes et al. studied the postnatal development of mice overexpressing this constitutively active form of CnA [46]. After one week these mice develop cardiac hypertrophy with reduced protein and RNA levels of Cx43 and Na_v_1.5. After three weeks the protein levels of Cx43 were still reduced and the connexin proteins were less phosphorylated, potentially as a result of dephosphorylation by CnA. Four weeks after birth these hearts display substantial levels of fibrosis, so increased levels of active CnA coincide with reduced expression of the sodium channel Na_v_1.5, Cx43 and eventually end in cardiac hypertrophy and fibrosis formation [46]. In addition, a different transgenic mouse model harbouring an inducible constitutively active CnA gene confirmed the presented adverse cardiac remodelling, but also indicated reversibility upon block of CnA [47]. CnA inhibition in a murine CaMKII knockout model confirmed the main role of CnA in cardiac hypertrophy, associated with fibrosis formation, apoptosis and systolic dysfunctions.

## Conclusion and future perspective

**Fig. 2:**
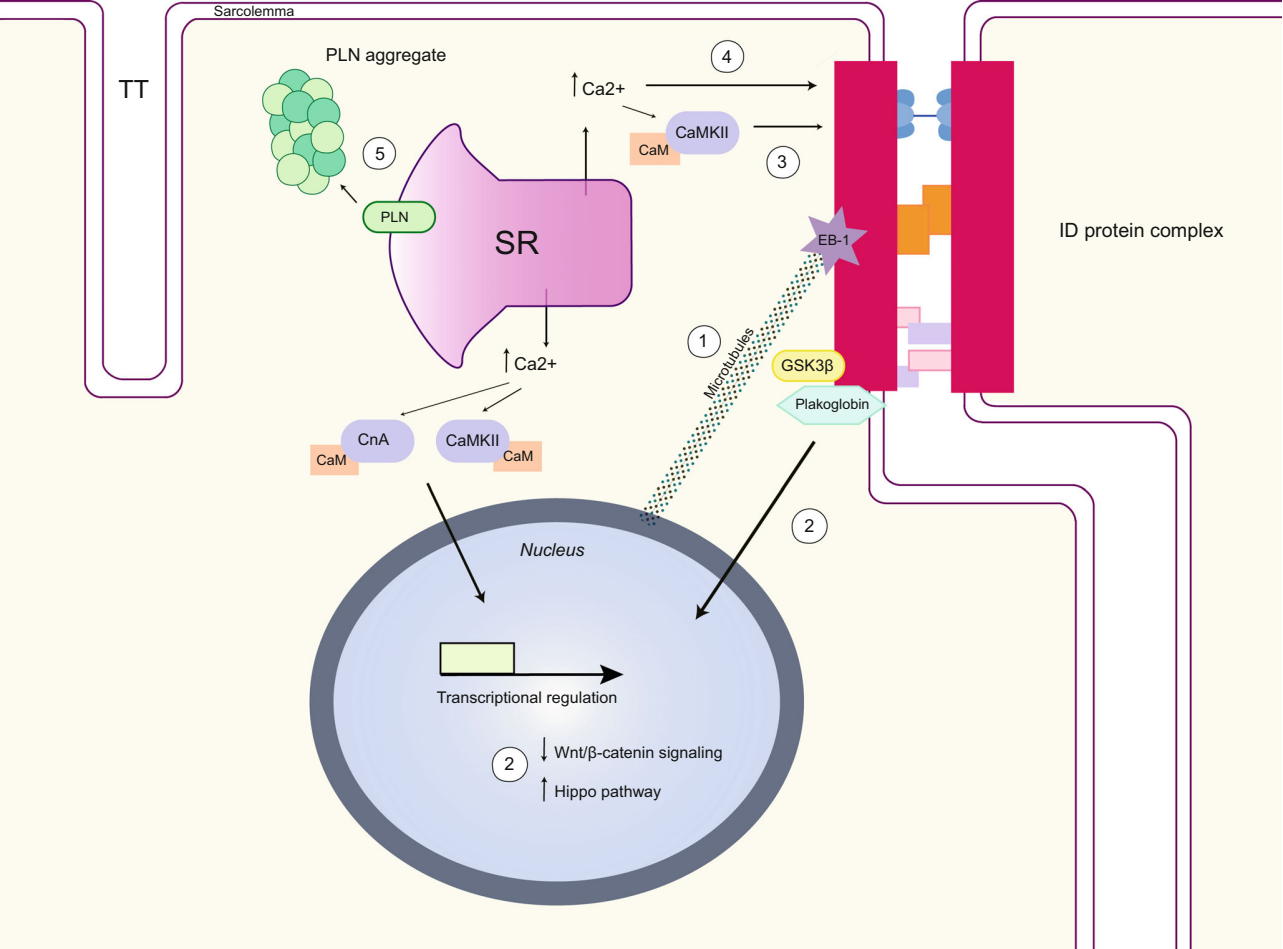
Figure illustrating five pathways putatively causing pathological remodelling in ACM: 1) Deficits in EB-1 ID protein trafficking provoke disturbance of the ID morphology. 2) Pathological activation of the hippo pathway and suppression of Wnt/β-catenin signalling, caused by molecular remodelling of ID proteins, Plakoglobin nuclear translocation and GSK3β-ID anchoring, recapitulates the ARVC phenotype. 3) Remodelling of ID proteins by Ca^2+^-activated CaMKII. 4) Possible direct effect of increased intracellular Ca^2+^ concentrations on the ID structure. 5) Intracellular accumulation and degradation of misfolded PLN proteins in PLN-R14Del mutation carriers. *GSK3*β glycogen synthase kinase 3 beta

ACM, or its most prominent subform ARVC, is commonly regarded as a disease of the ID in which mutations in desmosomal proteins are an important causative factor. Originally described as a composition of separate units, recent data indicate that the ID should be considered a single functional unit in which Nav1.5, Cx43, ankyrin-G and components of the adherence junction and desmosome (including PKP2 and plakoglobin) interact. As such, accumulating evidence in the last decade, suggests that destabilisation of the ID (through disturbed protein-protein interactions) and trafficking problems of its composing proteins trigger the remodelling. The latter aspect was recently illustrated through deficits in EB-1 ID protein trafficking causing a disturbed ID morphology resulting from a disrupted connection between the tubulin network (which directs the new proteins to the ID) and the existing macromolecular complex at the ID [48]. Other contributing mechanisms have recently also acquired more attention as detrimental roles are anticipated for intracellular accumulation of misfolded or mislocalised protein aggregates, and alterations in the hippo/Wnt signalling pathways. For the latter the specific contribution of GSK3β that accumulates at the ID, and plakoglobin that dispatches from the ID are topics of investigation (Fig. 2).

The Dutch founder mutation *PLN R14Del* has been identified to play a major role in ACM (at least in the Netherlands) which seems, as explained, remarkable since PLN is an important protein involved in regulation of SR calcium load. Despite of that, patients with this mutation show, beyond clinical signs that fit with ARVC, also remodelling of proteins in the ID and fibrofatty replacement. The mutation results in dysfunction of PLN and thereby accumulation of diastolic calcium, which in turn would be able to activate Ca^2+^ sensitive proteins as CaMKII and CnA. Patients with a different PLN mutation, *PLN-R25C*, indeed show activation of CaMKII which in the past has been linked to DCM, heart failure and disturbed calcium handling. As has been reported for patients with desmosomal mutations, dysfunction of the cardiac sodium channels is a rather recent observation. Although the experimental proof is still lacking, if this dysfunction results in enhanced *I*_Na-L_, also under these conditions enhanced diastolic calcium levels can be expected and as such activation of the described Ca^2+^-sensitive pathways. Activated CaMKII likely modifies ID proteins, SR structures and ion channels in a direct fashion, where CnA rectifies their transcriptional regulation and stimulates formation of fibrosis. In addition, enhanced cytosolic Ca^2+^ concentrations can induce autophagy by activating CaMKII, a manifestation also seen in PLN-R14Del patients [49]. Obviously, further experimental investigation is needed to unravel the exact role of CaMKII and CnA in ACM.

Step by step, important accomplishments have been made in our knowledge and understanding. Progression, however, is sometimes compromised by the fact that the applied experimental models do not always completely recapitulate the situation seen in patients. Obviously patient material is extremely scarce and often reflects the situation in the end stage only. Examples of the hurdles that adhere to experimental models are the fact that calcium handling in mouse cardiomyocytes, and in cardiomyocytes derived from iPS cells, is significantly different from that in humans [50]. Also the important aspect of fibro-fatty replacement, one of the hallmarks of the disease, is often only partially recapitulated in mouse models (mostly only the deposition of fibrosis). Ongoing improvements in experimental approaches and implementation of new techniques (e. g. focusing on genetic repair and application of super-resolution microscopy) provide, however, enough fuel and hope for a fruitful progression of our aim to control this cardiac disease.

**Table 1:** Schematic overview of PLN patient mutations, the experimental models used and findings obtained regarding the cardiac pathological remodelling in human and animals

PLN Mutation	Species	Mechanism	Effect			
*PLN-R9C mutation *[30]	*Human*	–	DCM, HF	Ventricular dilatation	Decreased cardiac output	Premature death
[30]	*Mice*	Trapped PKA & PKA-mediated PLN-P	Delayed calcium transients	DCM, HF	Premature death	–
*PLN-L39 STOP mutation *[31]	*Human*	Reduced PLN mRNA	No PLN protein	Hypertrophy, DCM, HF	–	–
[31]	*HEK cells & Adult Rat CM*	No SERCA inhibition by PLN	No stable PLN expression	PLN misrouted to cytosol & PM	–	–
*PLN-R14Del mutation *[11]	*Human*	–	Myocardial disarrangement	Fibrofatty replacement	ARVC	Premature death
*PLN-R14Del mutation (±)* [10]	*HEK cells*	Super inhibition SERCA	Pentamer destabilisation	Enhanced PLN-SERCA	DCM	Premature death
*PLN-R14Del overexpression *[10]	*Mice*	–	Dilatation LVW	Contractile dysfunction	Ventricular arrhythmias	–
[29]	*IPS-CM*	Abnormal distribution PLN to cytoplasm	Electrical instability	–	–	–
*PLN-R14Del mutation (−/−)* [21]	*Mice*	PLN-SERCA interaction/inhibition lost	Misrouted towards PM	NKA stimulation	Ventricular dilation & fibrosis	–
*PLN-R25C mutation* [32]	*Human*	–	DCM	Ventricular arrhythmias	Need for ICD implantation	–
[32]	*Rat*	Increased SERCA-PKA interaction	Decreased calcium transient	Increased Ca^2+^/CaMKII interaction	HyperP-RYR/Increased SR calcium	Increased diastolic calcium & VA

*PLN* phospholamban, *DCM* dilated cardiomyopathy, *HF* heart failure, *PKA* protein kinase A, *mRNA* messenger RNA, *PLN-P* phosphorylated PLN, *HEK cells* human embryonic kidney cells, *CM* cardiomyocytes; *SERCA* sarcoplasmic reticulum Ca^2+^-ATPase, *PM* plasma membrane, *ARVC* arrhythmogenic right ventricular cardiomyopathy, *LVW* left ventricular wall, *iPS-CM* induced pluripotent stem cell-derived cardiomyocytes, *NKA* Na^+^/K^+^-ATPase, *ICD* implantable cardioverter-defibrillator, *CaMKII* Ca^2+^/calmodulin-dependent protein kinase II, *HyperP-RYR* hyper-phosphorylated ryanodine receptor, *VA* ventricular arrhythmias
